# Exploratory analysis of a phase III trial of pirfenidone identifies a subpopulation of patients with idiopathic pulmonary fibrosis as benefiting from treatment

**DOI:** 10.1186/1465-9921-12-143

**Published:** 2011-10-28

**Authors:** Arata Azuma, Yoshio Taguchi, Takashi Ogura, Masahito Ebina, Hiroyuki Taniguchi, Yasuhiro Kondoh, Moritaka Suga, Hiroki Takahashi, Koichiro Nakata, Atsuhiko Sato, Shoji Kudoh, Toshihiro Nukiwa, Pirfenidone Clinical Study Group in Japan

**Affiliations:** 1 Division of Pulmonary Medicine, Infection and Oncology, Nippon Medical School, Tokyo, Japan; 2 Dept. of Respiratory Medicine, Tenri Hospital, Tenri, Japan; 3 Dept. of Respiratory Medicine, Kanagawa Cardiovascular and Respiratory Center, Yokohama, Japan; 4 Dept. of Respiratory Medicine, Tohoku University Graduate School of Medicine, Sendai, Japan; 5 Respiratory Medicine and Allergy, Tosei General Hospital, Aichi, Japan; 6 Dept. of Respiratory Medicine, Saiseikai Kumamoto Hospital, Kumamoto, Japan; 7 Third Dept. of Internal Medicine, Sapporo Medical University Hospital, Sapporo, Japan; 8 Nakata Clinic, Tokyo, Japan; 9 Kyoto Preventive Medical Center, Kyoto, Japan

## Abstract

**Background:**

A phase III trial in Japan showed that pirfenidone is effective for idiopathic pulmonary fibrosis (IPF). To find out which patients specifically benefit from pirfenidone, we analyzed in an exploratory manner the data from the phase III trial.

**Methods:**

The patients in the phase III trial were stratified by baseline percentage predicted vital capacity (%VC), arterial oxygen partial pressure (PaO_2_), and the lowest oxygen saturation by pulse oximetry (SpO_2_) during the 6-minute steady-state exercise test (6MET). In the subpopulations, changes in VC and subjective symptoms (cough and dyspnea on the Fletcher, Hugh-Jones [F, H-J] Classification scale) were evaluated in patients treated with high-dose (1800 mg/day) pirfenidone, low-dose (1200 mg/day) pirfenidone, and placebo at week 52.

**Results:**

Significant efficacy of pirfenidone in reducing the decline in VC could be seen in a subpopulation having %VC ≥ 70% and SpO_2 _< 90% at baseline. This favorable effect was accompanied by categorical change in VC and progression-free survival time. In the subpopulation, pirfenidone significantly suppressed cough and dyspnea.

**Conclusions:**

IPF patients having %VC ≥ 70% and SpO_2 _< 90% at baseline will most likely benefit from pirfenidone when evaluated using changes in VC (and %VC), and cough and dyspnea symptoms. This subpopulation could expect to benefit most from pirfenidone treatment.

**Trial Registration:**

This clinical trial was registered with the Japan Pharmaceutical Information Center (JAPIC) on September 13th, 2005 (Registration Number: JAPICCTI-050121).

## Background

Idiopathic pulmonary fibrosis (IPF) is a fatal, progressive fibrotic lung disease with a median survival of 3-5 years and no proven effective therapy to date [1,2]. Pirfenidone (5-methyl-1-phenyl-2-[1H]-pyridone; Shionogi & Co., Ltd., Osaka, Japan; Marnac Inc., Dallas, TX, USA) [3-7] is an antifibrotic drug for IPF which has combined anti-inflammatory, antioxidant, and antifibrotic effects in experimental models of pulmonary fibrosis [8-12]. A randomized, double-blind, placebo-controlled phase II trial of pirfenidone with 107 Japanese IPF patients demonstrated that pirfenidone significantly reduced the decline in vital capacity (VC) at week 36 compared to placebo (p = 0.037) [7]. These encouraging results prompted us to undertake a phase III clinical trial with 275 Japanese patients. In the Phase III trial, pirfenidone showed significant reduction in the decline of VC at week 52 (p = 0.042) and improved progression-free survival (PFS) time (p = 0.028) [13]. These Phase II and III data led to regulatory approval of pirfenidone in Japan for the treatment of IPF in 2008.

The Phase II trial in Japan also led to two larger, international, randomized trials of pirfenidone for IPF (CAPACITY, study 004 and study 006 with 435 and 344 IPF patients, respectively) [14]. In study 004, the decline in percentage predicted forced vital capacity (FVC) at week 72 was significantly reduced in pirfenidone-treated patients compared to those treated with placebo (p = 0.001). In study 006, the difference in FVC change at week 72 was not significant. However, the decline in %FVC was reduced at all time points during the first year. An analysis of pooled data from the two studies supported the treatment effect of pirfenidone on the %FVC, PFS time, and 6-minute walk test (6MWT) distance. In February 2011, pirfenidone was granted marketing authorization by the European Commission for the treatment of IPF.

Extended analyses of the phase III trial data in Japan revealed a subpopulation of patients who benefited from pirfenidone. Ebina et al. [15,16] examined the association between pirfenidone efficacy (changes in VC at week 52) and the baseline %VC, and reported that pirfenidone was more effective in patients with relatively mild impairment of lung function (%VC ≥70).

To clarify more precisely which patients specifically benefit from pirfenidone, we examined the association between pirfenidone efficacy and the baseline lung functions including %VC, arterial oxygen partial pressure (PaO_2_), and the lowest oxygen saturation in the 6-minute steady-state exercise test (the lowest SpO_2_). In each subgroup, the change in VC, PFS time, and subjective symptoms (cough and dyspnea on the Fletcher, Hugh-Jones [F, H-J] Classification scale) were evaluated after high-dose (1800 mg/day) pirfenidone, low-dose (1200 mg/day) pirfenidone, and placebo treatment for 52 weeks.

## Methods

### Overall Design

The phase III trial in Japan was a multicenter, double-blind, randomized, placebo-controlled trial. The diagnosis of IPF was in accordance with the American Thoracic Society (ATS)/European Respiratory Society (ERS) Consensus statement [17] and the "Clinical diagnostic criteria for idiopathic interstitial pneumonia (IIP)" (4th edition) in Japan [18]. Patients received either high-dose pirfenidone (1800 mg/day), low-dose pirfenidone (1200 mg/day), or placebo for 52 weeks.

The trial was conducted in accordance with the principles laid down in the Declaration of Helsinki (2002 version). The protocol was approved by the institutional review board at each center and the written informed consent was obtained from all participants prior to enrollment. The ongoing efficacy and safety results were reviewed by the independent the Data and Safety Monitoring Board (DSMB).

### Inclusion criteria

Eligible patients were adults (20-75 years old) with IPF diagnosed on the basis of the above criteria and meeting the following SpO_2 _criteria: 1) oxygen desaturation of >5% difference between the resting SpO_2 _and the lowest SpO_2_; 2) lowest SpO_2 _of ≥85% while breathing air.

### Patients and randomization

In all, 325 patients were screened at 73 centers in Japan, and 275 patients were randomized to one of three treatment groups (i.e., the high-dose, low-dose, and placebo groups) at a ratio of 2:1:2. Ultimately, 267 (108, 55, and 104 patients in high-dose, low-dose and placebo groups, respectively) were evaluated for the efficacy as the full analysis set (FAS). Eight patients were excluded for having no post-baseline data.

### Measurements

The measurements of VC, the lowest SpO_2_, PaO_2 _at rest, and subjective symptoms (cough and dyspnea intensity rated using the F, H-J classification system [19]) were defined as in our previous report [13]. The cough severity was rated either 1 [none; no cough], 2 [mild; intermittent cough], 3 [moderate; irritating, but not debilitating cough], and 4 [heavy; debilitating cough characterized by shortness of breath and exhaustion]. Progression-free survival (PFS) was defined by death and/or ≥10% decline in VC from baseline. VC was measured every 4 weeks, while the lowest SpO_2 _and other pulmonary function tests were observed every 12 weeks. In this trial, the primary endpoint was the change in VC. As the secondary endpoints, we used: 1) PFS time and 2) change in the lowest SpO_2 _during the 6-minute steady-state exercise test (6MET). Initially, the primary endpoint was the lowest SpO_2 _during the 6MET, as in the phase II trial [7]. Then, as explained in our previous report [13], a decision was made to change the primary endpoint to VC prior to breaking the code, in accordance with the recommendation of the DSMB.

### Statistical analysis

To identify the subpopulation that benefited most from pirfenidone treatment, we stratified the patients from the phase III trial using 70% of baseline %VC or 70 torr of baseline PaO_2 _and 90% of baseline SpO_2 _as boundary values. Namely, patients were stratified by baseline %VC (<70 vs ≥70%) or PaO_2 _(<70 vs ≥70) and the lowest SpO_2 _(<90 vs ≥90%). We selected these boundary values based on the results of the phase II trial in Japan and exploratory examination of the phase III trial by Ebina et al. [15].

Following analyses were performed in each of the subpopulations. Means of the changes in VC and %VC from baseline were compared between the treated (pirfenidone high-dose and low-dose) and placebo groups with the analysis of covariance (ANCOVA) using the respective baseline measurements as covariates. In the ANCOVA, the principle of the last observation carried forward (LOCF) was adopted to impute missing values. The cumulative PFS rates were estimated using the Kaplan-Meier method and the distributions of PFS time were compared using the log-rank test. ANCOVA was used to compare the means of the changes in subjective symptoms (i.e., cough and dyspnea scored on the F, H-J classification scale) between groups treated with either high- or low-dose pirfenidone or placebo. In these exploratory analyses, the significance level of tests was set at 0.1 (two-sided), inasmuch as 0.1 was the level used in the phase III study [13].

## Results

The phase III trial showed that pirfenidone reduced the decline in VC at week 52 in IPF patients, and significantly prolonged the PFS time, compared to placebo [13]. In this exploratory analysis, patients were grouped by baseline %VC or PaO_2 _at rest and the lowest SpO_2 _to identify the subpopulations that benefited most from pirfenidone treatment. Specifically, patients were stratified on the basis of %VC (<70 vs ≥70), PaO_2 _(<70 vs ≥70), and the lowest SpO_2 _(<90 vs ≥90).

### The changes in VC and %VC in subpopulations stratified by baseline %VC, PaO_2_, and the lowest SpO_2_

When patients were stratified by baseline %VC (<70%, ≥70%) and the lowest SpO_2 _(<90, ≥90), pirfenidone tended to be more effective in patients with baseline %VC ≥70 and the lowest SpO_2 _<90 (Subgroup A) than in the other subgroups. Namely, in Subgroup A, mean declines of VC and %VC at week 52 (all p-values <0.1**; **Table 1) and the distribution of progression-free survival times **(data not shown) **were significantly different between those treated with pirfenidone (high-dose, low-dose, and high+low dose) and those treated with placebo. When patients were stratified by PaO_2 _(<70, ≥70) and by SpO_2 _on exertion (<90, ≥ 90), pirfenidone tended to be more effective in patients with PaO_2 _≥70 and SpO_2 _on exertion <90 (Subgroup B) than in the other subgroups. P-values of the comparisons of mean declines in VC (%VC) between the treatment groups (pirfenidone high-dose, low-dose, and high+low dose) and the placebo group were 0.151 (0.198), 0.088 (0.097) and 0.059 (0.074), respectively **(**Table 2). In Subgroup B, a similar trend was seen in progression-free survival time **(data not shown)**.

**Table 1:** Decline in VC and %VC at week 52 in subpopulations characterized by baseline %VC and the lowest SpO_2_

Item	Category 1 SpO_2_	Category 2 Baseline %VC	High-dose Group		Low-dose Group		Placebo Group		P-value		
						
			LS mean (n)	SE	LS mean (n)	SE	LS mean (n)	SE	H vs P	L vs P	H+L vs P
Change in VC	6MWT SpO_2_≥90	%VC≥70	-0.072 (35)	0.033	-0.004 (16)	0.050	-0.090 (32)	0.035	0.7035	0.1595	0.2581
		
		%VC<70	-0.263 (9)	0.073	-0.185 (6)	0.090	-0.225 (9)	0.073	0.7181	0.7350	0.9919
	
	6MWT SpO_2_<90	%VC≥70	-0.050 (36)	0.049	-0.016 (17)	0.071	-0.199 (36)	0.049	0.0359	0.0372	0.0131
		
		%VC<70	-0.148 (23)	0.051	-0.181 (15)	0.062	-0.168 (26)	0.048	0.7768	0.8735	0.9539

Change in %VC	6MWT SpO_2_≥90	%VC≥70	-2.083 (35)	1.070	-0.420 (16)	1.580	-2.801 (32)	1.114	0.6438	0.2209	0.2927
		
		%VC<70	-9.084 (9)	2.446	-7.340 (6)	2.963	-7.167 (9)	2.449	0.5902	0.9647	0.7427
	
	6MWT SpO_2_<90	%VC≥70	-1.737 (36)	1.569	-0.590 (17)	2.264	-6.080 (36)	1.568	0.0555	0.0493	0.0213
		
		%VC<70	-4.143 (23)	1.637	-5.090 (15)	2.079	-5.669 (26)	1.567	0.5037	0.8280	0.6158

**Table 2:** Decline in VC and %VC at week 52 in subpopulations characterized by baseline PaO2 at rest and the lowest SpO2

Item	Category 1 SpO_2_	Category 2 PaO_2_	High-dose Group		Low-dose Group		Placebo Group		P-value		
						
			LS mean (n)	SE	LS mean (n)	SE	LS mean (n)	SE	H vs P	L vs P	H+L vs P
Change in VC	6MWT SpO_2_≥90	PaO_2_≥70 Torr	-0.115 (43)	0.032	-0.060 (22)	0.045	-0.137 (36)	0.035	0.6423	0.1766	0.2667
		
		PaO_2_<70 Torr	- (1)	-	- (0)	-	- (5)	-	-	-	-
	
	6MWT SpO_2_<90	PaO_2_≥70 Torr	-0.115 (42)	0.043	-0.087 (28)	0.053	-0.199 (56)	0.038	0.1509	0.0881	0.0585
		
		PaO_2_<70 Torr	-0.014 (15)	0.067	-0.102 (4)	0.129	-0.135 (6)	0.105	0.34560	0.8456	0.5556

Change in %VC	6MWT SpO_2_≥90	PaO_2_≥70 Torr	-3.486 (43)	1.013	-2.329 (22)	1.412	-4.544 (36)	1.107	0.4833	0.2200	0.2484
		
		PaO_2_<70 Torr	- (1)	-	- (0)	-	- (5)	-	-	-	-
	
	6MWT SpO_2_<90	PaO_2_≥70 Torr	-3.755 (42)	1.379	-2.629 (28)	1.698	-6.124 (5)	1.199	0.1975	0.0965	0.0744
		
		PaO_2_<70 Torr	-0.204 (15)	2.040	-3.038 (4)	3.951	-3.964 (6)	3.229	0.3364	0.8578	0.5568

(-; not calculated.)

**Figure 1:**
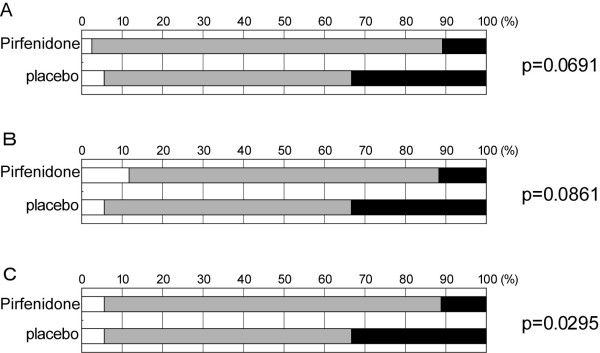
**Categorical changes in VC from baseline to week 52 in Subgroup A [baseline %VC ≥ 70 and the lowest SpO_2 _< 90]**. A) High-dose vs Placebo groups, B) Low-dose vs Placebo groups, C) pirfenidone-treated (High + Low-dose) vs placebo groups. (The changes in VC are rated as follows: improved, VC ≥ 10% increase; stable, VC < 10% change; worsened, VC ≥ 10% decline). The white, gray, and black areas indicate improvement, stability, and deterioration, respectively. The p-values of Wilcoxon rank sum test are indicated on the right.

Incidentally, in the phase II trial in Japan, the change in VC was categorized as "improved," "stable," or "deteriorated" in accordance with the ATS/ERS criteria [17] (where separation into three categories was based on 10% change in VC). The categorical change was significantly different between the pirfenidone treatment group and placebo group in the phase II trial but not in the phase III trial as described in the Online Supplementary Materials of our preceding paper [13]. By the way, patients in Subgroup B met the criteria for entry into the phase II trial, and Subgroup B was expected to resemble Subgroup A. Therefore, it was expected that significant differences might be seen by comparing categorical changes in Subgroup A and/or Subgroup B. Thus, the distributions of categorical changes in VC at week 52 were compared between pirfenidone (high- and low-dose) and placebo groups with the Wilcoxon rank sum test in "Subgroup A" and "Subgroup B" of the phase III trial. Then, changes in VC were classified as "improved," "stable," or "deteriorated" using the 10% change criterion employed in the Phase II trial. The differences in the distribution of categorical change in VC were statistically significant between high-dose and placebo groups (p = 0.0691), low-dose and placebo groups (p = 0.0861), and pooled (high + low) dose and placebo groups in Subgroup A (p = 0.0295**; **Figure 1) but not in "Subgroup B" **(data not shown)**.

### Temporal changes of subjective symptoms in subpopulations

**Figure 2:**
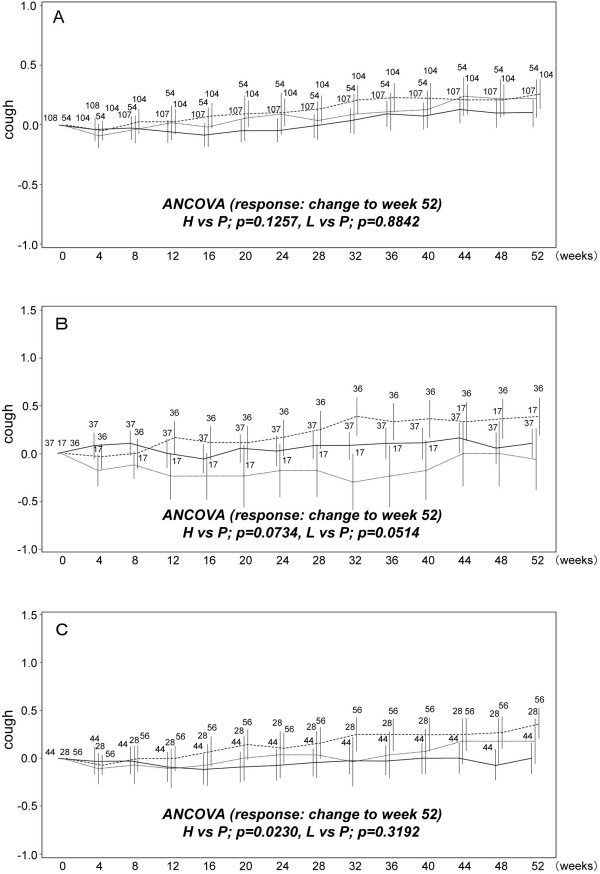
**Temporal changes in cough score in subpopulations**. A) Full analysis set (FAS; all patients), B) Subgroup A [%VC ≥ 70 and the lowest SpO_2 _< 90], and C) Subgroup B [PaO2 ≥ 70 and the lowest SpO_2 _< 90]. Data are shown as mean ± SE. High-dose (solid line); low dose (dashed line); placebo (dashed line in bold). The mean changes from baseline to week 52 were compared between high (or low-dose) and placebo groups with ANCOVA.

**Figure 3:**
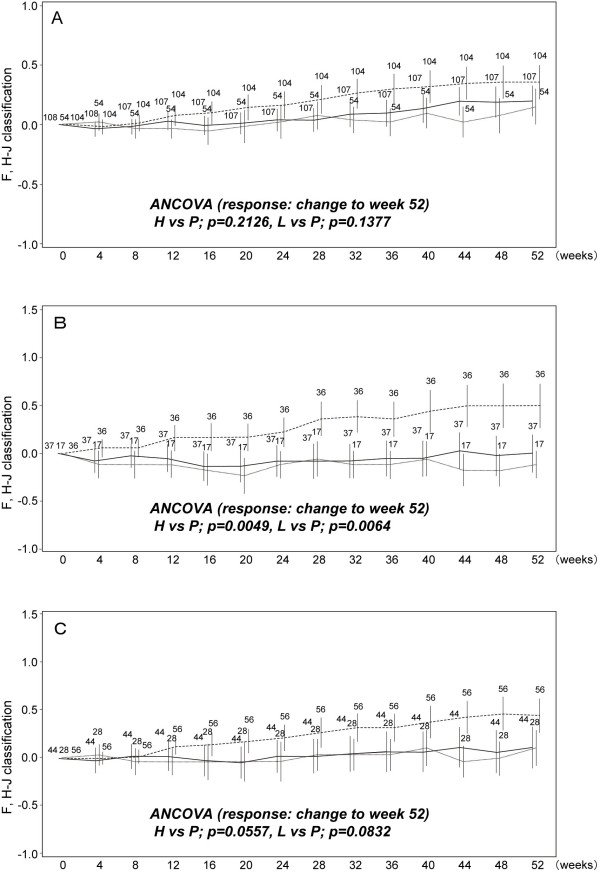
**Temporal changes in dyspnea score (F, H-J classification) in subpopulations**. A) Full analysis set (FAS; all patients), B) Subgroup A [%VC ≥ 70 and SpO_2 _during 6MET < 90], and C) Subgroup B [PaO2 ≥ 70 and SpO_2 _during 6MET < 90]. Data are shown as mean ± SE. High-dose (solid line); low dose (dashed line); placebo (dashed line in bold). The mean changes from baseline to week 52 were compared between high or low-dose and placebo groups with ANCOVA.

To evaluate the changes in subjective symptoms (i.e., cough and dyspnea) during the phase III trial, means of the changes in cough score and dyspnea score (with F, H-J classification) from baseline were calculated at each observation time and are shown in Figures 2 and 3. Further, the results of ANCOVA using the changes in cough and dyspnea scores from baseline to week 52 as responses, also are shown in Figures 2 and 3, respectively. In the full analysis set (FAS), pirfenidone tended to prevent the elevation of these scores more consistently in the high-dose and low-dose groups than in the placebo group, although the differences were not significant. When examined in Subgroups A and B, changes in cough and dyspnea scores showed that pirfenidone prevented increase in cough (Figure 2) and dyspnea (Figure 3) more effectively in Subgroups A and B than in the FAS at week 52. In addition, the differences in dyspnea scores seen in Subgroup A between pirfenidone (high- and low-dose) and placebo groups were significant at week 12 (p-values were 0.0428 and 0.0379, respectively). The significant difference in cough score in Subgroup A was seen between low-dose and placebo group (p = 0.0502).

## Discussion

Our exploratory analyses using changes in VC, categorical changes in VC, PFS time, and scores on subjective symptoms as outcomes suggested that pirfenidone was more effective in patients with mild-to-moderate lung function impairment (baseline %VC ≥ 70 and the lowest SpO_2 _< 90; Subgroup A). In addition, pirfenidone had significant effects on some of these outcomes in Subgroup B (baseline PaO_2 _≥ 70 and the lowest SpO_2 _< 90). In the population of patients with mild-to-moderate disease, pirfenidone is especially effective in patients with desaturation during exercise which typically corresponds to the lowest baseline SpO_2 _< 90.

To evaluate temporal changes in subjective symptoms in the phase III trial, means of the changes in cough and in F, H-J classification scores were calculated. In FAS, pirfenidone tended to prevent the elevation of these scores more consistently in high- and low-dose groups than in the placebo group, but no significant differences were detected. Additional analysis in the present study, however, showed that compared to placebo, pirfenidone significantly prevented elevation of these scores at week 52 and significantly lowered dyspnea score as early as week 12 especially in Subgroup A. These results suggest that pirfenidone can be expected to prevent worsening of subjective symptoms such as dyspnea and exert this effect at a very early stage in the same patient population shown to have reduced impairment of respiratory functions such as VC. We additionally compared the incidence of acute exacerbation between pirfenidone and placebo groups in Subgroup A. The incidence in the pirfenidone group (1.82% [1/55]) was lower than that in the placebo group (8.33% [3/36]), although the difference was not statistically significant (data not shown).

In the 'responsive' subgroup, i.e., patient subgroup with baseline %VC of ≥70 and the lowest SpO_2 _of <90, lung function is better preserved though oxygen saturation is deteriorated. These are mutually conflicting characteristics. As pulmonary fibrosis progresses at the early stage of the disease, interstitial septa between the alveoli and capillaries, which are the major sites of gas exchange, become thickened and gas exchange and diffusion become impaired. Hypoxemia is thought to be caused by pulmonary-diffusion impairment and ventilation-perfusion ratio mismatching. The ventilation-perfusion ratio mismatching is more likely during exercise due to dynamic change of pulmonary circulation and increased flow, irrespective of PaO_2 _status at rest [20]. In this way, the thickening of the lung interstitial septa leads to desaturation and dyspnea during exercise at an early stage of the disease progression. The results showing that pirfenidone tended to be more effective in patients with %VC ≥70 and the lowest SpO_2 _<90 than in patients with %VC <70 and the lowest SpO_2 _<90 (Table 1) suggest that the desaturation exhibited by the subgroup with more preserved lung function may have been due to developing fibrosis with inflammatory edema and not to established fibrosis. Pirfenidone could be more effective in treating such 'young' fibrosis and therefore able to inhibit progression of fibrosis during the early stage. Furthermore, pirfenidone, which also has anti-inflammatory activity [3], may be effective in improving dyspnea by reducing inflammatory edema and vascular permeability. Above explanations are speculative, and therefore a future prospective clinical study is necessary to confirm the better response to this drug by this subgroup.

Recent clinical trials of drugs for IPF tend to include mild-to-moderate cases while totally excluding severe cases, as in the clinical trials of IFN-γ [21]. This is probably because the inclusion of severe cases can bias the evaluation of treatment effectiveness. In the phase II trial of pirfenidone for Japanese patients [7], mean decline in VC was 130 mL in the placebo group and 30 mL in pirfenidone-treated groups with a p-value of 0.0356 at week 36. The reduction in decline by pirfenidone was greater than that obtained in the phase III trial in Japan [13]. The entry criteria of the phase II trial included PaO_2 _at rest of ≥70 torr and the lowest SpO_2 _during 6MET of ≤90%, while criteria of the phase III trial were broader (i.e., 1] oxygen desaturation of >5% difference between resting SpO_2 _and the lowest SpO_2_, and 2] the lowest SpO_2 _>85% while breathing air). These characteristics were similar in patients enrolled in the phase II trial and in the Subgroup B patients of the phase III trial enrolled in this study. Pirfenidone was shown to have a more marked effect on both the patients with the lowest SpO_2 _≤ 90% in Subgroup B as those in the phase II trial (data not shown). Possibly, the phase II trial might have enrolled a population of patients who were more responsive to the drug. The broader criteria for inclusion into the phase III trial might have resulted in a more heterogeneous population and more variable data.

In Japan, the severity of idiopathic interstitial pneumonia (IIPs) is classified on the basis of baseline PaO_2 _value at rest, and categories are defined by 10-torr intervals. The grade of IIPs in patients with SpO_2 _of < 90 on exertion is increased by one (except for grade I) as described in the online supplemental materials of our previous report of the Phase III trial [13]. In the phase III trial, patients were grouped based on severity to identify the subpopulation that was more responsive to pirfenidone. Pirfenidone was found to be more effective in grade-III patients **(data not shown)**. A more detailed analysis revealed that the population of patients with PaO_2 _≥ 70 and < 80 included many grade-III patients when SpO_2 _was < 90% on exertion **(data not shown)**. These findings also may support the efficacy of pirfenidone in patients with desaturation during exercise.

Identifying those patients clinically responsive to pirfenidone is very important. The present analyses revealed that pirfenidone was more effective in populations of patients with relatively favorable baseline %VC and PaO_2_, especially in those with desaturation on exertion. Since pirfenidone was more effective in Subgroup A than in Subgroup B, baseline %VC may be a more appropriate index than PaO_2_. In addition, patients presenting desaturation during exercise may be comparable to those complaining of dyspnea on exertion. For more beneficial use of pirfenidone, the factors--baseline %VC and the presence/absence of complaints of dyspnea on exertion--may be used to select candidate patients. However, since responsiveness in this study depended on the stage of the disease as determined by respiratory function tests, these factors cannot be regarded as indicative of a responsive phenotype but rather as indicative of a responsive "phenostage" (coinage by the authors). (A 'Phenotype' determining response to therapy, for example to anti-cancer therapy, is generally characterized by expression of a specific gene, whereas the 'responsiveness' of the subgroup identified in this study may be due to the timing of treatment during disease progression rather than a specific gene.) A sub-analysis of data from the CAPACITY trials [14] yielded similar results. The FVC change at week 72 showed that a subpopulation of patients given oxygen during 6MWT at baseline responded favorably to pirfenidone [22]. To determine whether this observation and our findings are equivalent, a detailed sub-analysis of data from the CAPACITY trials or further prospective studies will be needed.

To support the results obtained from the analyses described in preceding sections, we used respiratory function tests at baseline to determine the factors associated with the efficacy of pirfenidone. Thus, we included percentage predicted total lung capacity (%TLC), %DLco in addition to the lowest SpO_2_, %VC, and PaO_2_. Then, we used the change in VC from baseline to week 52 as the efficacy parameter and evaluated the effects of the 5 function tests on this efficacy parameter in pirfenidone and placebo groups. At first, correlation coefficients among the 5 respiratory tests were calculated in the pirfenidone and placebo groups. The correlation coefficients between %VC and %TLC were very high in both groups (0.811 and 0.826, respectively). Thus, we subsequently omitted %TLC from the evaluation, and retained %VC since %VC behaves like VC (which was the primary endpoint in the phase III trial) and was considered indispensable in the additional analysis. Then, we applied a multiple regression model letting the change in VC serve as the response variable and the four respiratory function tests as explanatory variables in the two groups (Table 3). From the Tables, the regression coefficient of %VC in the pirfenidone group was significant (p = 0.0018), and it was suggested that in patients with relatively low baseline %VC, the tendency to prevent the decline in VC was greater in the pirfenidone group than in the placebo group.

**Table 3:** Effects of respiratory tests on the change in VC in Pirfenidone and Placebo groups

Group	Parameter	Estimate	**S.E**.	t-value	p-value
Pirfenidone	Intercept	-0.3543	0.7428	-0.48	0.6340
(n = 155)	The lowest SpO_2_	0.0029	0.0091	0.32	0.7514
	%VC	0.0035	0.0011	3.17	0.0018
	%DLco	-0.0011	0.0011	-0.97	0.3361
	PaO_2_	-0.0025	0.0021	-1.19	0.2378

Placebo	Intercept	-2.0951	1.2726	-1.65	0.1029
(n = 102)	The lowest SpO_2_	0.0217	0.0146	1.48	0.1412
	%VC	0.0008	0.0017	0.49	0.6279
	%DLco	-0.0017	0.0017	-0.99	0.3248
	PaO_2_	0.0003	0.0033	0.10	0.9172

(%TLC was omitted from the evaluation)

Further, three dichotomized variables (the lowest SpO_2_, %VC, and PaO_2 _with boundary values of 90%, 70%, and 70 torr, respectively) were used in the stratification, and the effects of the variables on the change in VC were evaluated with a multiple regression model. In the pirfenidone group, the coefficients of %VC and PaO_2 _were significant (p-values, 0.0002 and 0.0483, respectively, see Table 4). In the placebo group, the coefficients were not significant. This seems to support the findings presented in the previous sections, namely that the most favorable response to pirfenidone relative to placebo was in patients with %VC ≥70% and SpO_2 _<90 (Subgroup A). Notably, when patients were stratified using 70% as the boundary value of %VC, decline in VC was reduced in patients with %VC ≥70 after pirfenidone treatment but not after placebo treatment. In addition, although the coefficient of SpO_2 _was not significant, the decline of VC in patients with SpO_2 _<90% tended to be relatively small in the pirfenidone group and large in the placebo group. Accordingly, patients with %VC ≥70% and SpO_2 _<90 (Subgroup A) received more benefit from pirfenidone than did other patients. For patients with PaO2 ≥70% and <70, the change in VC differed less between the pirfenidone and placebo groups as indicated by the negative signs of both regression coefficients of the dichotomized PaO_2_. Therefore, it was suggested that efficacy of pirfenidone was less clear in Subgroup B than in Subgroup A.

**Table 4:** Effects of respiratory function tests (values dichotomized) on the change in VC in pirfenidone and placebo groups

Group	Parameter	Estimate	**S.E**.	t-value	p-value
Pirfenidone	Intercept	-0.0857	0.0523	-1.64	0.1039
(n = 155)	The lowest SpO_2_: <90 vs ≥90	-0.0090	0.0381	-0.24	0.8133
	%VC: <70 vs ≥70	0.1447	0.0386	3.75	0.0002
	PaO_2_: <70 vs ≥70	-0.1111	0.0558	-1.99	0.0483

Placebo	Intercept	-0.1196	0.0923	-1.30	0.1982
(n = 103)	The lowest SpO_2_: <90 vs ≥90	0.0628	0.0566	1.11	0.2701
	%VC: <70 vs ≥70	0.0302	0.0585	0.52	0.6067
	PaO_2_: <70 vs ≥70	-0.0977	0.0878	-1.11	0.2685

## Limitations

Given the exploratory nature of our study, limitations include post-hoc analysis and small sample size due to stratification. As a consequence, our findings should be considered preliminary and need to be confirmed in future studies. However, the phase II and III trials in Japan as well as the CAPACITY trials identified VC or FVC as a promising endpoint to judge the efficacy of treatment for IPF [14]. The baseline respiratory functions used to select patients for entry into the phase II trial are incidentally similar to those of patients benefiting from drug treatment in the phase III trial. In addition, the following limitations should be considered.

In this study, missing values were imputed with the LOCF method. Collard [23] as well as Swigris and Fairclough [24] pointed out the problems associated with using the LOCF method. Indeed, the method is simple but has its own deficiencies. However, there are no perfect imputation methods, and we explained the issues in detail as a rejoinder to Swigris and Fairclough [25].

In this paper, we analyzed subjective symptom outcomes or "patient-reported outcomes." Though, the results of analyses on these outcomes were not shown in the original paper as pointed by Collard [23]. The reason was that we didn't place much value in cough and dyspnea (F, H-J classification) as patient-reported outcomes, since these outcomes were treated as tertiary end-points in the phase III trial. In addition, these outcomes were observed not retrospectively but prospectively, as was described in section "Efficacy end-points" of our previous paper [13].

Additional clinical studies are desired to confirm the findings obtained in this study.

## Conclusion

The results of these explanatory analyses identified IPF patients having baseline %VC ≥ 70 and SpO_2 _< 90% during 6MET as the subpopulation that benefited most from pirfenidone treatment in terms of the changes in VC, PFS, and subjective symptoms such as cough and dyspnea. It is suggested that this subpopulation, especially, will benefit from pirfenidone treatment.

## Abbreviations used in this paper

IPF: idiopathic pulmonary fibrosis; VC: vital capacity; PFS: progression-free survival; SpO_2_: oxygen saturation by pulse oximetry; %DLco: % diffusing capacity of the lung for carbon monoxide; %TLC: % predicted total lung capacity; FAS: full analysis set; PFT: pulmonary function test; 6MET: 6-minute steady-state exercise test; ANCOVA: analysis of covariance; LOCF: last observation carried forward; ATS: American Thoracic Society; ERS: European Respiratory Society; F: H-J, Fletcher, Hugh-Jones; DSMB: Data and Safety Monitoring Board.

## Competing interests

AA, YT, ME, HT, MS, HT, KN, AS, SK, and TN have received consultancy fees for advisory board activities, and AA, TO, ME, YK, HT and TN have received fees for speaking from Shionogi & Co., Ltd.

## Authors' contributions

All authors listed made significant conceptual and intellectual contributions to the design and conception of the study, substantially contributed to the article, and have provided final approval of the version submitted. The members of Pirfenidone Clinical Study Group in Japan contributed as the principal investigator at each center.
